# Improving Automated Essay Scoring by Prompt Prediction and Matching

**DOI:** 10.3390/e24091206

**Published:** 2022-08-29

**Authors:** Jingbo Sun, Tianbao Song, Jihua Song, Weiming Peng

**Affiliations:** 1 School of Artificial Intelligence, Beijing Normal University, Beijing 100875, China; 2 School of Computer Science and Engineering, Beijing Technology and Business University, Beijing 100048, China

**Keywords:** automated essay scoring, natural language processing, multi-task learning, pre-trained language model, hierarchical structure model

## Abstract

Automated essay scoring aims to evaluate the quality of an essay automatically. It is one of the main educational application in the field of natural language processing. Recently, Pre-training techniques have been used to improve performance on downstream tasks, and many studies have attempted to use pre-training and then fine-tuning mechanisms in an essay scoring system. However, obtaining better features such as prompts by the pre-trained encoder is critical but not fully studied. In this paper, we create a prompt feature fusion method that is better suited for fine-tuning. Besides, we use multi-task learning by designing two auxiliary tasks, prompt prediction and prompt matching, to obtain better features. The experimental results show that both auxiliary tasks can improve model performance, and the combination of the two auxiliary tasks with the NEZHA pre-trained encoder produces the best results, with Quadratic Weighted Kappa improving 2.5% and Pearson’s Correlation Coefficient improving 2% on average across all results on the HSK dataset.

## 1. Introduction

Automated essay scoring (AES), which aims to automatically evaluate and score essays, is one typical application of natural language processing (NLP) technique in the field of education [1]. In earlier studies, a combination of handcrafted design features and statistical machine learning is used [2,3], and with the development of deep learning, neural network-based approaches gradually become mainstream [4,5,6,7,8]. Recently, pre-trained language models have gradually become the foundation module of NLP, and the paradigm of pre-training, then fine-tuning, is also widely adopted. *Pre-training* is the most common method for transfer learning, in which a model is trained on a surrogate task and then adapted to the desired downstream task by *fine-tuning* [9]. Some research has attempted to use pre-training modules in AES tasks [10,11,12]. Howard et al. [10] utilize the pre-trained encoder as a feature extraction module to obtain a representation of the input text and update the pre-trained model parameters based on the downstream text classification task by adding a linear layer. Rodriguez et al. [11] employ a pre-trained encoder as the essay representation extraction module for the AES task, with inputs at various granularities of the sentence, paragraph, overall, etc., and then use regression as the training target for the downstream task to further optimize the representation. In this paper, we fine-tune the pre-trained encoder as a feature extraction module and convert the essay scoring task into regression as in previous studies [4,5,6,7].

The existing neural methods obtain a generic representation of the text through a hierarchical model using convolutional neural networks (CNN) for word-level representation and long short-term memory (LSTM) for sentence-level representation [4], which is not specific to different features. To enhance the representation of the essay, some studies have attempted to incorporate features such as prompt [3,13], organization [14], coherence [2], and discourse structure [15,16,17] into the neural model. These features are critical for the AES task because they help the model understand the essay while also making the essay scoring more interpretable. In actual scenarios, prompt adherence is an important feature in essay scoring tasks [3]. The hierarchical model is insensitive to changes in the corresponding prompt for the essay and always assigns the same score for the same essay, regardless of the essay prompt. Persing and Ng [3] propose a feature-rich approach that integrates the prompt adherence dimension. Ref. [18] improves document modeling with a topic word. Li et al. [7] utilizes a hierarchical structure with an attention mechanism to construct prompt information. However, the above feature fusion methods are unsuitable for fine-tuning.

The two challenges in effectively incorporating pre-trained models into AES feature representation are the data dimension and the methodological dimension. For the data dimension, the use of fine-tuning approaches to transfer the pre-trained encoder to downstream tasks frequently necessitates sufficient data, and there has been more research on both training and testing data from the same target prompt [4,5], but the data size is relatively small, varying between a few hundred and a few thousand, and pre-trained encoders cannot be fine-tuned well. In order to solve this challenge, we use the whole training set, which includes various prompts. In terms of methodology, we employ the pre-training and multi-task learning (MTL) paradigms, which can learn features that cannot be learned in a single task through joint learning, learning to learn, and learning with auxiliary tasks [19], etc. MTL methods have been applied to several NLP tasks, such as text classification [20,21], semantic analysis [22] et al. Our method creates two auxiliary tasks that need to be learned alongside the main task. The main task and auxiliary tasks can increase each other’s performance by sharing information and complementing each other.

In this paper, we propose an essay scoring model based on fine-tuning that utilizes multi-task learning to fuse prompt features by designing two auxiliary tasks, prompt prediction, and prompt matching, which is more suitable for fine-tuning. Our approach can effectively incorporate the prompt feature in essays and improve the representation and understanding of the essay. The paper is organized as follows. In Section 2, we first review related studies. We describe our method and experiment in Section 3 and Section 4. Section 5 presents the findings and discussions. Finally, in Section 6, we provide a conclusion, future work, and the limitations of the paper.

## 2. Related Work

Pre-trained language models, such as BERT [23], BERT-WWM [24], RoBERTa [25], and NEZHA [26], have gradually become a fundamental technique for NLP, with great success on both English and Chinese tasks [27]. In our approach, we use the BERT and NEZHA feature extraction layers. BERT is the abbreviation of Bidirectional Encoder Representations from Transformers, and it is based on transformer blocks that are built using the attention mechanism [28] to extract semantic information. It is trained on two unsupervised tasks using large-scale datasets: masked language model (MLM) and next sentence prediction (NSP). NEZHA is a Chinese pre-training model that employs functional relative positional encoding and whole word masking (WWM) rather than BERT. The pre-training then the fine-tuning mechanism is widely used in downstream NLP tasks, including AES [11,12,15]. Mim et al. [15] propose a pre-training approach for evaluating the organization and argument strength of essays based on modeling coherence. Song et al. [12] present a multi-stage pre-training method for automated Chinese essay scoring that consists of three components: weakly supervised pre-training, supervised cross-prompt fine-tuning, and supervised target-prompt fine-tuning. Rodriguez et al. [11] use BERT and XLNET [29] for representation and fine-tuning of English corpus.

The essay prompt introduces the topic, offers concepts, and restricts both content and perspective. Some studies have attempted to enhance the AES system by incorporating prompt features in many ways, such as by integrating prompt information to determine if an essay is off-topic [13,18] or by considering prompt adherence as a crucial indicator [3]. Louis and Higgins [13] improve model performance by expanding prompt information with a list of related words and reducing spelling errors. Persing and Ng [3] propose a feature-rich method for incorporating the prompt adherence dimension via manual annotation. Klebanov et al. [18] also improve essay modeling with topic words to quantify the overall relevance of the essay to the prompt, and the relationship between prompt adherence scores and total essay quality is also discussed. The methods described above mostly employ statistical machine learning, prompt information is enriched by annotation and the construction of datasets, as well as the construction of word lists and topic word mining. While all of them are making great progress, the approaches they are employing are more difficult to directly transfer to fine-tuning. Li et al. [7] propose a shared model and an enhanced model (EModel), and utilize a neural network hierarchical structure with an attention mechanism to construct features of the essay such as discourse, coherence, relevancy, and prompt. For the representation, the paper employs GloVe [30] rather than a pre-trained model. In the experiment section, we compared our method to the sub-module of EModel (Pro.) which incorporates the prompt feature.

## 3. Methods

### 3.1. Motivation

**Figure 1:**
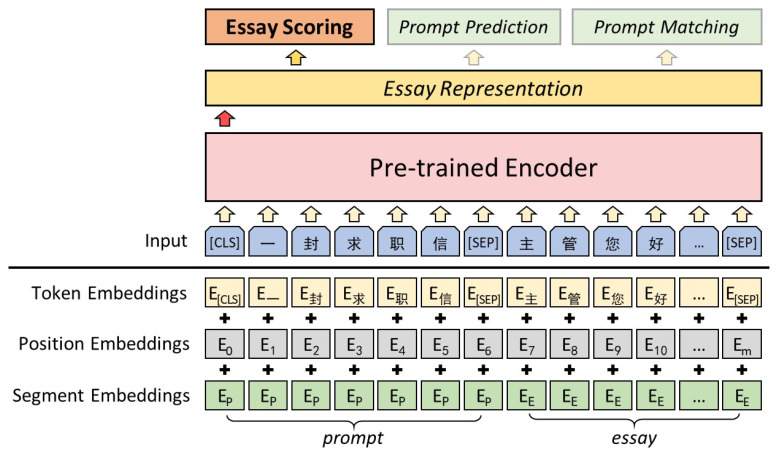
The proposed framework. “一封求职信” is the prompt of the essay, the English translation is “A cover letter”. “主管您好” means “Hello Manager”. The prompt and essay are separated by [SEP].

Although previous studies on automated essay scoring models for specific prompts have shown promising results, most research focuses on generic features of essays. Only a few studies have focused on prompt feature extraction, and no one has attempted to use a multi-task approach to make the model capture prompt features and be sensitive to prompts automatically. Our approach is motivated by capturing prompt features to make the model aware of the prompt and using pre-training and then the fine-tuning mechanism for AES. Based on this motivation, we use a multi-task learning approach to obtain features that are more applicable to *Essay Scoring (ES)* by adding essay prompts to the model input and proposing two auxiliary tasks: *Prompt Prediction* (*PP*) and *Prompt Matching* (*PM*). The overall architecture of our model is illustrated in Figure 1.

### 3.2. Input and Feature Extraction Layer

The input representation for a given essay is built by adding the corresponding token embeddings $E_{token}$, segment embeddings $E_{segment}$, and position embeddings $E_{position}$. To fully exploit the prompt information, we concatenate the prompt in front of the essay. The first token of each input is a special classification token [CLS], and the prompt and essay are separated by [SEP]. The token embedding of the *j*-th essay in the *i*-th prompt can be expressed as Equation (1), $E_{segment}$ and $E_{position}$ are obtained from the tokenizer of the pre-train encoder.

(1) $$E_{token}^{\left(i\right)\left(j\right)}=\left\{E_{prompt}^{\left(i\right)},E_{essay}^{\left(i\right)\left(j\right)}\right\}.$$

We utilize the BERT and NEZHA as feature extraction layers. The final hidden state corresponding to the [CLS] token is the essay representation $r_{e}$ for essay scoring and subtasks.

### 3.3. Essay Scoring Layer

We view essay scoring as a regression task. To enable data mapping regression problems, the real scores are scaled to the range [0,1] for training and rescaled during evaluation, according to the existing studies:

(2) $$s^{\left(i\right)\left(j\right)}=\frac{score^{\left(i\right)\left(j\right)}-min\left(score^{\left(i\right)}\right)}{max\left(score^{\left(i\right)}\right)-min\left(score^{\left(i\right)}\right)},$$

where $s^{\left(i\right)\left(j\right)}$ is the scaled score for *i*-th prompt *j*-th essay, and $score^{\left(i\right)\left(j\right)}$ is the actual score for *i*-th prompt *j*-th essay, $max\left(score^{\left(i\right)}\right)$ and $min\left(score^{\left(i\right)}\right)$ are the maximum and minimum of the real scores for the *i*-th prompt. The input is essay representation $r_{e}$ from the pre-trained encoder, which is fed into a linear layer with a sigmoid activation function:

(3) $$\hat{s}=\sigma \left(W_{es}\;\cdot \;r_{e}+b_{es}\right),$$

where $\hat{s}$ is the predicted score by AES system, σ is the sigmoid function, $W_{es}$ is a trainable weights, and $b_{es}$ is a bias. The essay scoring (es) training objective is described as:

(4) $$\ell oss_{es}\left(s,\hat{s}\right)=\frac{1}{N}\sum_{k}^{N}{\left(s^{k}-{\hat{s}}^{k}\right)}^{2}.$$

### 3.4. Subtask 1: Prompt Prediction

The definition of prompt prediction is giving an essay to determine which prompt it belongs to. We view prompt prediction as a classification task. The input is essay representation $r_{e}$, which is fed into a linear layer with a softmax function. The formula is given by Equation (5):

(5) $$\hat{u}=\mathrm{softmax}\left(W_{pp}\;\cdot \;r_{e}+b_{pp}\right),$$

where $\hat{u}$ is the probability distribution of classification results, $W_{pp}$ is a parameter matrix, and $b_{pp}$ is a bias. The loss function is formalized as follows:

(6) $$\begin{array}{cc} \ell oss_{pp}\left(u,\hat{u}\right)\; & =-\frac{1}{N}\sum_{k}^{N}\sum_{c=1}^{C}f\left(u^{\left(k\right)},c\right)\log\left(p_{pp}^{\left(k\right)\left(c\right)}\right), \end{array}$$

(7) $$\begin{array}{cc} f(x,y) & =\left\{\begin{array}{ccc} 1\; & if & x=y\\ 0\; & else & x\neq y \end{array}\;,\right. \end{array}$$

where $u^{\left(k\right)}$ is the real prompt label for the *k*-th sample, $p_{pp}^{\left(k\right)\left(c\right)}$ is the probability that the *k*-th sample belongs to the *c*-th category, *C* denotes the number of prompts, which in this study is ten.

### 3.5. Subtask 2: Prompt Matching

The definition of prompt matching is giving a pair of a prompt and an essay, and to decide if the essay and the prompt are compatible. We consider prompt matching to be a classification task. The following is the formula:

(8) $$\hat{v}=\mathrm{softmax}\left(W_{pm}\;\cdot \;r_{e}+b_{pm}\right),$$

where $\hat{v}$ is the probability distribution of matching results, $W_{pm}$ is a parameter matrix, and $b_{pm}$ is a bias. The objective function is shown in Equation (9)

(9) $$\ell oss_{pm}\left(v,\hat{v}\right)=-\frac{1}{N}\sum_{k}^{N}\sum_{m=0}^{M}f\left(v^{\left(k\right)},m\right)\log\left(p_{pm}^{\left(k\right)\left(m\right)}\right),$$

where $v^{\left(k\right)}$ indicates whether the input prompt and essay match. $p_{pm}^{\left(k\right)\left(m\right)}$ is the likelihood that the matching degree of *k*-th sample falls into category m. *m* denotes the matching degree, 0 for a match, 1 for a dismatch. The distinction between prompt prediction and prompt matching is that as the number of prompts increases, the difference in classification targets leads to increasingly obvious differences in task difficulty, sample distribution and diversity, and scalability.

### 3.6. Multi-Task Loss Function

The final loss function for each input is a weighted sum of the loss functions for essay scoring and two subtasks: prompt prediction and prompt matching, with the loss formalized as follows:

(10) $$\ell oss_{MTL}=\alpha \;\cdot \;\ell oss_{es}+\beta \;\cdot \;\ell oss_{pp}+\gamma \;\cdot \;\ell oss_{pm},$$

where α, β, and γ are non-negative weights assigned in advance to balance the importance of the three tasks. Because the objective of this research is to improve the AES system, the main task should be given more weight than the two auxiliary tasks. The optimal parameters in this paper are α:β=α:γ= 100:1, and in Section 5.3, we design experiments to figure out the optimal value interval for α, β, and γ.

## 4. Experiment

### 4.1. Dataset

We use HSK (HSK is the acronym of Hanyu Shuiping Kaoshi, which is Chinese Pinyin for the Chinese Proficiency Test). Dynamic Composition Corpus (http://hsk.blcu.edu.cn/ (accessed on 6 March 2022)) as our dataset as in existing studies [31]. HSK is also called “TOEFL in Chinese”, which is a national standardized test designed to test the proficiency of non-native speakers of Chinese. The HSK corpus includes 11,569 essays composed by foreigners from more than thirty different nations or regions in response to more than fifty distinct prompts. We eliminate any prompts with fewer than 500 student writings from the HSK dataset to constitute the experimental data. The statistical results of the final filtered dataset are provided in Table 1, which comprises 8878 essays across 10 prompts taken from the actual HSK test. Each essay score ranges from 40 to 95 points. We divide the entire dataset at random into the training set, validation set, and test set in the ratio of 6:2:2. To alleviate the problem of insufficient data under a single prompt, we apply the entire training set that consists of different prompts for fine-tuning. We test every prompt individually as well as the entire test set during the testing phase and utilize the same 5-fold cross-validation procedure as [4,5]. Finally, we report the average performance.

### 4.2. Evaluation Metrics

For the main task, we use the Quadratic Weighted Kappa (QWK)approach, which is widely used in AES [32], to analyze the agreement between prediction scores and the ground truth. QWK can be calculated by Equations (11) and (12)

(11) $$W_{i,j}=\frac{{\left(i-j\right)}^{2}}{{\left(N-1\right)}^{2}},$$

where *i* and *j* are the golden score of the human rater and the AES system score, and each essay has *N* possible ratings. Second, calculate the QWK score using Equation (12).

(12) $$\mathrm{QWK}=1-\frac{\sum_{i,j}W_{i,j}O_{i,j}}{\sum_{i,j}W_{i,j}Z_{i,j}},$$

where $O_{i,j}$ denotes the number of essays that receive a rating *i* by the human rater and a rating *j* by the AES system. The expected rating matrix Z is histogram vectors of the golden rating and AES system rating and normalized so that the sum of its elements equals the sum of its elements in O. We also utilize Pearson’s Correlation Coefficient (PCC) to measure the association as in previous studies [3,32,33], which quantifies the degree of linear dependency between two variables and describes the level of covariation. In contrast to the QWK metric, which evaluates the agreement between the model output and the gold standard, we use PCC to assess whether the AES system ranks essays similarly to the gold standard, indicating the capacity of the AES system to appropriately rank texts, i.e., high scores ahead of low scores. For auxiliary tasks, we consider prompt prediction and prompt matching as classification problems and use macro-F1 score (F1), and accuracy (Acc.) as evaluation metrics.

### 4.3. Comparisons

Our model is compared to the baseline models listed below. The former three are existing neural AES methods, and we experiment with both character and word input when training for comparison. The fourth method is to fine-tune the pre-trained model, and the rest are variations of our proposed method.

**CNN-LSTM [4]:** This method builds a document using CNN for word-level representation and LSTM for sentence-level representation, as well as the addition of a pooling layer to obtain the text representation. Finally, the score is obtained by applying the linear layer of the sigmoid function.

**CNN-LSTM-att [5]:** This method incorporates an attention mechanism into both the word-level and sentence-level representations of CNN-LSTM.

**EModel (Pro.):** This method concatenates the prompt information in the input layer of CNN-LSTM-att, which is a sub-module of [7].

**BERT/NEZHA-FT:** This method is used to fine-tune the pre-trained model. To obtain the essay representation, we directly feed an essay into the pre-trained encoder as the input. We choose the [CLS] embedding as essay representations and feed them into a linear layer of the sigmoid function for scoring.

**BERT/NEZHA-concat:** The difference between this method and fine-tune is that the input representation concatenates the prompt to the front of the essay in token embedding, as in Figure 1.

**BERT/NEZHA-PP:** This model incorporates prompt prediction as an auxiliary task, with the same input as the concat model and the output using [CLS] as the essay representation. A linear layer with the sigmoid function is used for essay scoring, and a linear layer with the softmax function is used for prompt prediction.

**BERT/NEZHA-PM:** This model includes prompt matching as an auxiliary task. In the input stage of constructing the training data, there is a 50% probability that the prompt and the essay are mismatched. [CLS] embedding is used to represent the essay. A linear layer with the sigmoid function is used for essay scoring, and a linear layer with the softmax function is used for prompt matching.

**BERT/NEZHA-PP&PM:** This model utilizes two auxiliary tasks, prompt prediction, and prompt matching, with the same inputs and outputs as the PM model. The output layer of the auxiliary tasks is the same as above.

### 4.4. Parameter Settings

We use BERT (https://github.com/google-research/bert (accessed on 11 March 2022)) and NEZHA (https://github.com/huawei-noah/Pretrained-Language-Model/tree/master/NEZHA-TensorFlow (accessed on 11 March 2022)) as pre-trained encoder. To obtain tokens and token embeddings, we employ the tokenizer and vocabulary of the pre-trained encoder. The parameters of the pre-trained encoder are learnable during both the fine-tuning and training phases. The maximum length of the input is set to 512 and Table 2 includes additional parameters. The baseline models, CNN-LSTM and CNN-LSTM-att, are trained from scratch, and their parameters are shown in Table 2. Our experiments are carried out on NVIDIA TESLA V100 32 G GPUs.

## 5. Results and Discussions

### 5.1. Main Results and Analysis

We report our experimental results in Table 3 and Table A1 (Due to space limitations, this table is included in Appendix A). Table A1 illustrates the average QWK and PCC for each prompt. Table 3 shows QWK and PCC across the entire test set and the average results of each prompt test set. As shown in Table 3, we can find that the proposed auxiliary tasks (PP, PM, and PP&PM) (line 8–10 & 13–15) outperform other contrast models on both QWK and PCC, PP&PM models with the pre-trained encoder, BERT, and NEZHA, outperform PP and PM on QWK. In terms of the PCC metric, PM models exceeded the other two models except for the average result with the NEZHA encoder. The findings above indicate that our proposed two auxiliary tasks are both effective.

On *Total* test set, our best results, a pre-trained encoder with PM and PP, are higher compared to *fine-tuning* method and EModel(Pro.), exceed the strong baseline *concat* model by 1.8% with BERT and 2.3% with NEZHA on QWK, and get a generally consistent correlation. It is shown from Table 3 that our proposed models also yield similar results to the *Average* test set, 1.6% of BERT and 2% of NEZHA on QWK of PP&PM models compared to *concat* model, 2% of BERT and 2.5% of NEZHA on QWK of PP&PM models compared to *fine-tuning* model, and competitive results on PCC metric. Using the multi-task learning approach and *fine-tuning* comparison, our proposed approach outperforms the baseline system on both QWK and PCC, indicating that better essay representation can be obtained through multi-tasking learning. Furthermore, when compared to the *concat* model with fused prompt representation, our proposed approach outperform the baseline in QWK scores, but line 10 and line 15 in Table 3 *Total* track PCC values are lower within 1% of the baseline. It demonstrates that our proposed auxiliary task is effective in representing the essay prompt.

We train the hierarchical model (line 1–4) using character and word as input, respectively, and the results show that using the character for training is generally better, with the best results in *Total* and *Average* being more than 4% lower than those with the pre-training method. The results indicate that using pre-trained encoders both BERT and NEZHA for feature extraction works well on the HSK dataset. The pre-training model comparison reveals that BERT and NEZHA are competitive, with NEZHA delivering the best results.

**Figure 2:**
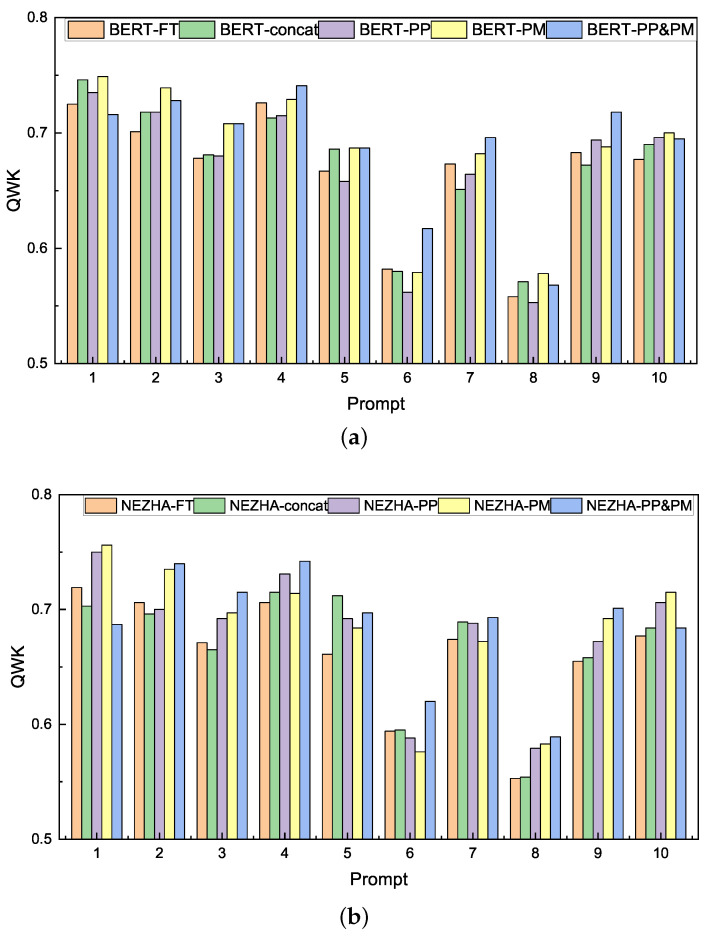
(**a**) Results of each prompt with BERT pre-trained encoder on QWK; (**b**) Results of each prompt with NEZHA pre-trained encoder on QWK.

Results of each prompt with BERT and NEZHA are displayed in Figure 2. The results of our proposed models (PP, PM, and PP&PM) have made positive progress on several prompts. Among them, the results of PP&PM, in addition, to prompt 1 and prompt 5, extend beyond the two baselines of *fine-tuning* and *concat*. The results indicate that our proposed auxiliary tasks to incorporate prompt is generic and can be employed with a range of genres and prompts. The primary cause of the results of individual prompts being suboptimal is that the hyperparameters of loss function α, β, and γ are not adjusted specifically for each prompt and we will further analyze the reasons for this in Section 5.3.

### 5.2. Result and Effect of Auxiliary Tasks

Table 4 depicts the results of the auxiliary tasks (PP and PM) on validation set, the accuracy and F1 are both greater than 85% for BERT and 90% for NEZHA, and the model is well trained in the auxiliary task, when compared to both pre-trained models BERT and NEZHA, the latter produces better. The results of auxiliary tasks with NEZHA perform better as feature extraction modules.

**Figure 3:**
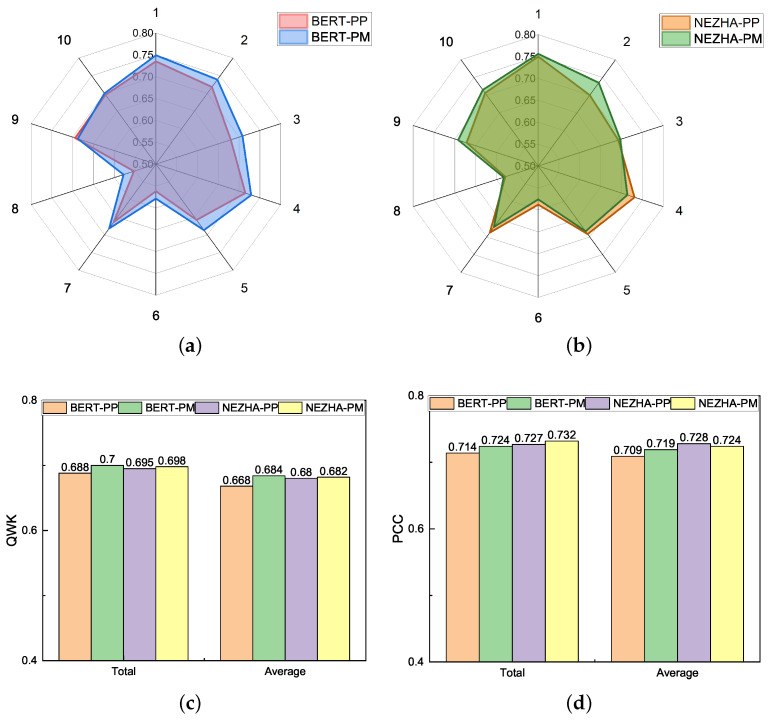
(**a**) Radar graph of BERT-PP&BERT-PM; (**b**) Radar graph of NEZHA-PP&NEZHA-PM; (**c**) Results of PP and PM on QWK; (**d**) Results of PP and PM on PCC.

Comparing the contribution of PP and PM, as shown in Table A1 and Table 3 and Figure 3, the contribution of PM is higher and more effective. Figure 3a,b illustrate radar graphs of various pre-trained encoders of PP and PM across 10 prompts utilizing QWK metrics. Figure 3a shows that the QWK value of PM is higher than PP in all but prompt 9 with BERT encoder, and Figure 3b demonstrates that the results of PM are 60% better compared to those of PP, implying that PM is also superior to PP for a specific prompt. The PM and PP comparison results for the *Total* and *Average* datasets are provided in Figure 3c,d. Except for the PM model with the NEZHA pre-trained encoder, which has a slightly lower QWK than the PP model, all models that use PM as a single auxiliary task perform better, further demonstrating the superiority of prompt matching in prompt representing and incorporating.

### 5.3. Effect of Loss Weight

**Figure 4:**
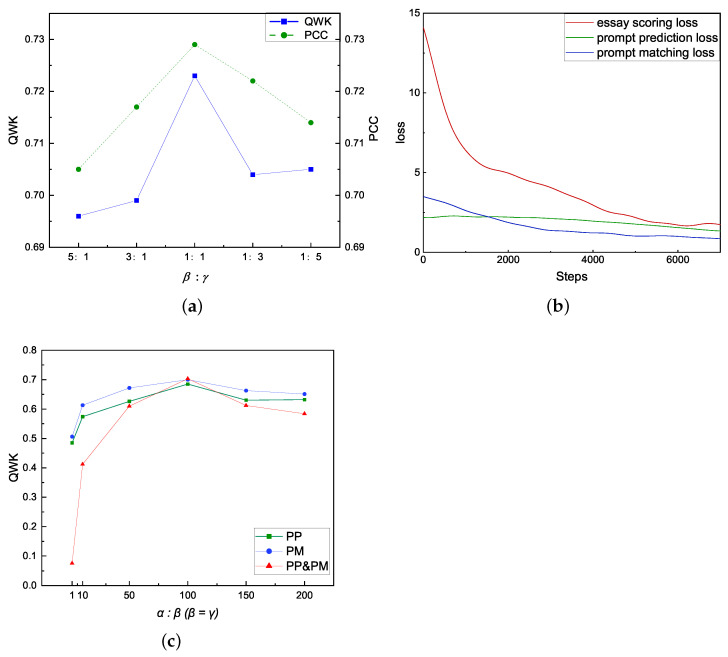
(**a**) The effect of PP&PM in different β/γ ratios of QWK and PCC on *Total* dataset, we fix the value of α in this section of the experiment.; (**b**) The smoothing results for training losses across all tasks; (**c**) The results of different α:β (PP), α:γ (PM), and α:β:γ (PP&PM) ratios on QWK.

**Figure A1:**
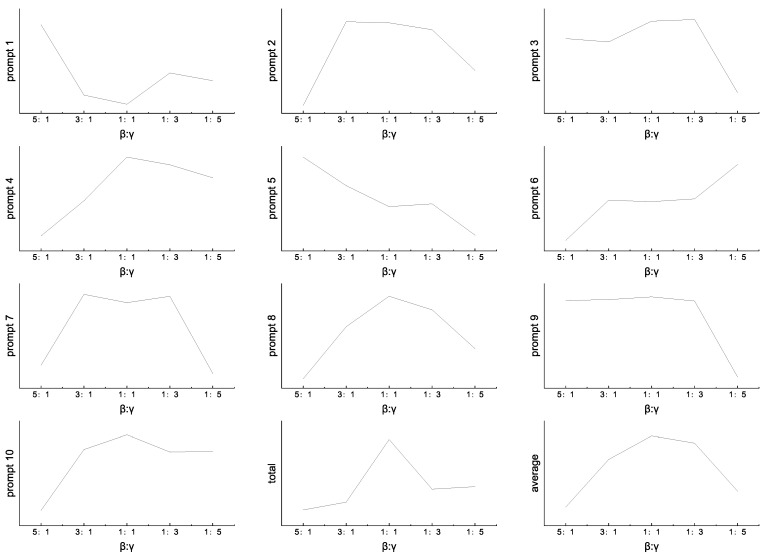
The effect of PP&PM in different β/γ ratios of QWK across all dataset, we fix the value of α in this section of the experiment.

We examine how the ratio of loss weight parameters β and γ affects the model. Figure 4a shows that the model works best when the ratio is 1:1 on both QWK and PCC metrics. Figure A1 depicts the QWK results for various β and γ ratios, as well as revealing that the model produces the greatest results at around 1:1 for different prompts, except for prompts 1, 5, and 6, and the same is true for the average results. Concerning the issue of our model being suboptimal for individual prompts, Figure A1 illustrates that the best results for prompts 1, 5, and 6 are not achieved at 1:1, suggesting that it is inappropriate for such parameters in these prompts. Because we disorder the entire training set and fix the β and γ ratio before testing it independently, the parameters of the different prompts cannot be dynamically adjusted within a single training procedure. The reasons are to address the lack of data and also to focus more on the average performance of the model, which also prevents the model from overfitting for specific prompts. Compared to the results in Table A1, NEZHA-PP and NEZHA-PM both outperform the baselines and the PP&PM model for prompt 1, indicating that both PP and PM can enhance the results when employed separately. For prompt 5, NEZHA-PP performs better than NEZHA-PM, showing that PP plays a greater role. The PP&PM model is already the best result for prompt 6, even though the 1:1 parameter is not optimal in Figure A1, demonstrating that there is still potential for improvement. The information above reveals that different prompts have varying degrees of difficulty for joint training and parameter optimization of the main and auxiliary tasks, along with different conditions of applicability for the two auxiliary tasks we presented.

We also measure the effect of α on the model, where we fix the β/γ ratio constant at 1:1. Figure 4c demonstrates that the PP, PM, and PP&PM models are all optimal at α:β=α:γ= 100:1, with the best QWK values for PP&PM, indicating that our suggested method of combining two auxiliary tasks for joint training is effective. The observation of [1,100] shows that when the ratio is small, the main task cannot be trained well, the two auxiliary tasks have a negative impact on the main task, but the single auxiliary task has less impact, indicating that multiple auxiliary tasks are more difficult to train concurrently than a single auxiliary task. In addition, future research should consider how to dynamically optimize the parameters of multiple tasks.

The training losses for ES, PP, and PM are included in Figure 4b, and it can be seen that the loss of the main task decreases rapidly in the early stage, and the model converges around 6000 steps. The reason for faster model convergence in PM is that the task is a dichotomous classification compared to PP, which is a ten classification, and additionally, among the ten prompts, prompt 6 “A letter to parent” and prompt 9 “Parents are children’s first teachers” are more similar, making PP more difficult. As a result, further research into how to select the appropriate weight ratio and design more matching auxiliary tasks is required.

## 6. Conclusions and Future Work

This paper presents a pre-training and then fine-tuning model for automated essay scoring. The model incorporates the essay prompts to the model input and obtains better features more applicable to essay scoring by multi-task learning with two auxiliary tasks, prompt prediction, and prompt matching. Experiments demonstrate that the model outperforms baselines in results measured by the QWK and PCC on average across all results on the HSK dataset, indicating that our model is substantially better in terms of agreement and association. The experimental results also show that both auxiliary tasks can effectively improve the model performance, and the combination of the two auxiliary tasks with the NEZHA pre-trained encoder yields the best results, with QWK enhancing 2.5% and PCC improving 2% compared to the strong baseline, the concatenate model, on average across all results on the HSK dataset. When compared to existing neural essay scoring methods, the experimental results show that QWK improves by 7.2% and PCC improves by 8% on average across all results.

Although our work has enhanced the effectiveness of the AES system, there are still limitations. Regarding the data dimension, this research primarily investigates fusing prompt features in Chinese; other languages are not examined extensively. Nevertheless, our method is more convenient for migration than the manual annotation approach, and other languages can be directly migrated. Furthermore, other features in different languages can use our method to create similar auxiliary tasks for information fusion. Moreover, as the number of prompts grows, the difficulty of training for prompt prediction increases, and we will consider combining prompts with genre and other information to design auxiliary tasks suitable for more prompts, as well as attempting to find a balance between the number of essays and the number of prompts to make prompt prediction more efficient. The parameters of the loss function are now defined empirically at the methodological level, which is not conducive to additional auxiliary activities. In future work, we will optimize the parameter selection scheme and build dynamic parameter optimization techniques to accommodate variable numbers of auxiliary tasks. In terms of application, our approaches focus on fusing textual information in prompts, while they do not cover all prompt forms. Our system now requires additional modules for the chart and picture prompt. In future research, we will experiment with multimodal prompt data to improve the application scenarios of the AES system.

## Figures and Tables

**Table 1:** HSK dataset statistic.

Set	#Essay	Avg #len	Chinese Prompt (English Translation)
1	522	336	一封求职信
			(A cover letter)
2	703	395	记对我影响最大的一个人
			(Remember the person who influenced me the most)
3	707	340	如何看待“安乐死”
			(How to view “euthanasia”)
4	957	338	由“三个和尚没水喝”想到的
			(Thought on “Three monks without water”)
5	829	356	如何解决“代沟”问题
			(How to solve the “generation gap”)
6	694	387	一封写给父母的信
			(A letter to parents)
7	1529	350	绿色食品与饥饿
			(Green food and hunger)
8	1333	330	吸烟对个人健康和公众利益的影响
			(Effects of smoking on personal health and public interest)
9	865	347	父母是孩子的第一任老师
			(Parents are children’s first teachers)
10	739	337	我看流行歌曲
			(My opinion on popular songs)

**Table 2:** Parameter settings.

Parameters	Baselines Settings	Our Methods Settings
Embedding size	100	768
Vocab size	500	21,128
Epoch	50	10
Batch size	64	16
Optimizer	RMSprop	Adam
Learning rate	1 × 10^−3^	5 × 10^−6^
LSTM hidden state	100	-
CNN filters (kernel size)	100 (5)	-
Word embedding	Tencent (small) https://ai.tencent.com/ailab/nlp/en/download.html (accessed on 17 March 2022)	-

**Table 3:** QWK and PCC for the total test set and Average QWK and PCC for each prompt test set; † denotes input as a character; ‡ denotes input as word. The best results are in bold.

Models	Total		Average	
	QWK	PCC	QWK	PCC
CNN-LSTM †	0.632	0.672	0.612	0.642
CNN-LSTM-att †	0.642	0.672	0.615	0.648
CNN-LSTM ‡	0.617	0.653	0.596	0.633
CNN-LSTM-att ‡	0.623	0.658	0.603	0.629
EModel (Pro.) ‡	0.642	0.669	0.620	0.649
BERT-FT	0.683	0.722	0.667	0.713
BERT-concat	0.685	0.719	0.671	0.712
BERT-PP	0.688	0.714	0.668	0.709
BERT-PM	0.700	**0.726**	0.684	**0.719**
BERT-PP&PM	**0.703**	0.711	**0.687**	0.715
NEZHA-FT	0.676	0.714	0.662	0.708
NEZHA-concat	0.681	0.717	0.667	0.714
NEZHA-PP	0.695	0.727	0.680	**0.728**
NEZHA-PM	0.698	**0.732**	0.682	0.724
NEZHA-PP&PM	**0.704**	0.714	**0.687**	0.722

**Table 4:** Accuracy and F1 for PP and PM on validation set.

Models	Prompt Prediction		Prompt Matching	
	Acc. (%)	F1 (%)	Acc. (%)	F1 (%)
BERT-PP&PM	86.6	85.6	85.5	85.6
NEZHA-PP&PM	91.7	98.1	90.7	91.4

## Data Availability

Publicly available datasets were used in this study. These data can be found here: http://hsk.blcu.edu.cn/ (accessed on 6 March 2022).

## Abbreviations

The following abbreviations are used in this manuscript:

AES	Automated Essay Scoring
NLP	Natural Language Processing
QWK	Quadratic Weighted Kappa
PCC	Pearson’s Correlation Coefficient

## Appendix A

**Table A1:** QWK and PCC for each prompt on HSK dataset, † denotes input as character; ‡ denotes input as word. The best results are in bold.

Metrics	QWK	PCC	QWK	PCC	QWK	PCC	QWK	PCC	QWK	PCC
**Method**	**Prompt 1**		**Prompt 2**		**Prompt 3**		**Prompt 4**		**Prompt 5**	
CNN-LSTM †	0.721	0.742	0.634	0.644	0.646	0.669	0.644	0.661	0.666	0.702
CNN-LSTM-att †	0.759	0.767	0.639	0.650	0.662	0.683	0.649	0.671	0.654	0.695
CNN-LSTM ‡	0.730	0.749	0.638	0.657	0.613	0.663	0.673	0.696	0.671	0.709
CNN-LSTM-att ‡	**0.767**	0.773	0.622	0.634	0.679	0.701	0.680	0.694	0.668	0.705
EModel (Pro.) ‡	0.752	0.769	0.664	0.681	0.672	0.687	0.693	0.710	0.676	0.704
BERT-FT	0.725	0.765	0.701	0.748	0.678	0.720	0.726	0.763	0.667	0.699
BERT-concat	0.746	0.772	0.718	0.756	0.681	0.726	0.713	0.751	0.686	**0.709**
BERT-PP	0.735	0.773	0.718	0.758	0.680	0.724	0.715	0.743	0.658	0.681
BERT-PM	**0.749**	0.774	**0.739**	**0.771**	**0.708**	**0.744**	0.729	**0.753**	**0.687**	0.704
BERT-PP&PM	0.716	**0.780**	0.728	0.766	**0.708**	0.734	**0.741**	**0.753**	**0.687**	0.707
NEZHA-FT	0.719	0.769	0.706	0.763	0.671	0.715	0.706	0.744	0.661	0.689
NEZHA-concat	0.703	0.751	0.696	0.761	0.665	0.715	0.715	0.754	**0.712**	**0.737**
NEZHA-PP	0.750	**0.791**	0.700	0.764	0.692	**0.747**	0.731	**0.763**	0.692	0.728
NEZHA-PM	**0.756**	0.787	0.735	**0.774**	0.697	0.741	0.714	0.760	0.684	0.717
NEZHA-PP&PM	0.687	0.781	**0.740**	0.765	**0.715**	0.745	**0.742**	0.761	0.697	0.710
**Method**	**Prompt 6**		**Prompt 7**		**Prompt 8**		**Prompt 9**		**Prompt 10**	
CNN-LSTM †	0.539	0.564	0.553	0.580	0.456	0.496	0.612	0.669	0.646	0.688
CNN-LSTM-att †	0.552	0.581	0.552	0.604	0.454	0.507	0.598	0.660	0.630	0.661
CNN-LSTM ‡	0.479	0.519	0.542	0.565	0.396	0.446	0.596	0.652	0.627	0.674
CNN-LSTM-att ‡	0.486	0.516	0.553	0.590	0.356	0.399	0.575	0.616	0.649	0.665
EModel (Pro.) ‡	0.503	0.528	0.560	0.602	0.413	0.457	0.597	0.661	0.667	0.693
BERT-FT	0.582	0.625	0.673	0.705	0.558	0.625	0.683	0.746	0.677	0.733
BERT-concat	0.580	**0.630**	0.651	0.698	0.571	**0.619**	0.672	0.720	0.690	0.738
BERT-PP	0.562	0.615	0.664	0.700	0.553	0.611	0.694	**0.739**	0.696	0.740
BERT-PM	0.579	0.620	0.682	**0.711**	**0.578**	**0.619**	0.688	0.736	**0.700**	**0.752**
BERT-PP&PM	**0.617**	0.627	**0.696**	0.705	0.568	0.601	**0.718**	**0.739**	0.695	0.741
NEZHA-FT	0.594	0.631	0.674	0.707	0.553	0.599	0.655	0.722	0.677	0.738
NEZHA-concat	0.595	0.642	0.689	0.718	0.554	0.610	0.658	0.716	0.684	0.738
NEZHA-PP	0.588	0.639	0.688	**0.723**	0.579	**0.633**	0.672	**0.745**	0.706	0.751
NEZHA-PM	0.576	0.630	0.672	0.719	0.583	0.624	0.692	0.740	**0.715**	**0.752**
NEZHA-PP&PM	**0.620**	**0.647**	**0.693**	0.715	**0.589**	0.618	**0.701**	0.729	0.684	0.750
